# Positive Aging in Demanding Workplaces: The Gain Cycle between Job Satisfaction and Work Engagement

**DOI:** 10.3389/fpsyg.2016.01224

**Published:** 2016-08-15

**Authors:** Dina Guglielmi, Lorenzo Avanzi, Rita Chiesa, Marco G. Mariani, Ilaria Bruni, Marco Depolo

**Affiliations:** ^1^Department of Educational Science, University of Bologna, BolognaItaly; ^2^Department of Psychology and Cognitive Science, University of Trento, TrentoItaly; ^3^Department of Psychology, University of Bologna, BolognaItaly

**Keywords:** engagement, aging, job satisfaction, gain cycle, job demands

## Abstract

Nowadays organizations have to cope with two related challenges: maintaining an engaged and highly performing workforce and, at the same time, protecting and increasing employees’ well-being and job satisfaction under conditions of a generalized increase of job demand, in an increasingly growing older population. According to the motivational process of the JD-R model, a work environment with many organizational resources will foster work engagement, which in turn will increase the likelihood of positive personal and organizational outcomes, such as job satisfaction, performance, and intention to stay. However, it is not clear how this motivational process could work in different age cohorts, as older workers may have different priorities to those of younger colleagues. Postulating the existence of a gain-cycle in the relationship between work engagement and outcomes, in this study we tested a longitudinal moderated mediation model in which job satisfaction increases over time through an increment in work engagement. We hypothesized that this process is moderated by job demand and aging. We collected data in public administrations in Northern Italy in order to measure work engagement and job satisfaction. 556 workers aged between 50 and 64 replied to the survey twice (the first time and 8 months later). The findings confirmed a moderated mediation model, in which job satisfaction at time 1 increased work engagement, which in turn fostered job satisfaction 8 months later, confirming the hypothesized gain-cycle. This relationship was shown to be moderated by the joint influence of job demand intensity and age: higher job demands and younger age are related to the maximum level of level gain cycle, while the same high level of job demands, when associated with older age, appears unable to stimulate a similar effect. The results confirm that, on one hand, older workers cannot be seen as a homogeneous group and, on the other hand, the importance of considering the role played by the gain cycle of resources. Our findings show that age matters, and that greater consideration should be devoted to age differences in order to design appropriate human resources practices that foster work engagement and satisfaction.

## Introduction

The changing workforce configuration is one of the most important challenges for the organizations of the new Millennium (Kompier, 2006). In particular, the aging population is a very relevant demographic variation that will lead to older workers playing a prominent role in the workforce in the near future.

In European Union countries, also due to the impact of “recent pension reforms in many Member States aimed at increasing the retirement age,” the employment rate of older workers (aged 55–64) is expected to increase continuously, shifting from 13.7% in 2013 to 18.3% in 2060 (European Commission, 2015, p. 65). As confirmed by the United Nations (2007, p. 24), by 2050 workers aged 50 years or over “are expected to make up almost one third (31.2%) of the working-age population in developed countries.” This scenario is complicated by other phenomena (i.e., internationalization, market deregulation, increased utilization of information and communication technology) that tend to change the nature of work, with the consequent increase in the percentage of workers experiencing high levels of stress (European Foundation for the Improvement of Living and Working Conditions, 2007). Although, work-related health problems also affect young employees (see Arcangeli and Mucci, 2009), this has become crucial as for elder workers.

This means that organizations have to cope with two related and important challenges: maintaining an engaged and highly performing workforce and, at the same time, protecting and increasing the employees’ well-being and job satisfaction under conditions of a generalized increase of job demand in an increasingly aging working population. Consequently, more attention should be paid to understanding how to motivate and satisfy older employees. In the past, several studies found a positive relationship between age and job satisfaction (Kalleberg and Loscocco, 1983; Bedeian et al., 1992; Ng and Feldman, 2007). More recently, a research study by the Sloan Center on Aging and Work at Boston College (2011) examined the work experiences of employees in 11 countries, confirming that those 50 years-old and older are the most engaged and satisfied with their jobs. However, little is known about the antecedents of older workers’ job satisfaction.

The aim of this study is to test the relationship between job satisfaction and engagement of older workers measured over time, considering the mediation of work engagement and the moderation of job demands and age.

We based our research on two important theoretical contributions: the Job Demands-Resources Model (JD-R; Bakker and Demerouti, 2007) and the Conservation of Resources Theory (COR; Hobfoll, 1989, 2011). According to the motivational process of the JD-R model, a work environment with many organizational resources (i.e., control or support) will foster a positive attitude toward work (engagement), which in turn will increase the likelihood of positive personal and organizational outcomes, such as job satisfaction, performance, and intention to stay. The added value of our research is that in addition to the effect of work engagement on job satisfaction, we expect to find a positive effect of job satisfaction on work engagement (with the moderation of job demand). This assumption is in line with the COR theory (Hobfoll, 2002), which postulated that people seek to obtain and accumulate resources in order to tackle stressful conditions. On one hand, people tend to prevent the loss of existing resources; on the other hand, they try to increase new personal and social resources, thus creating the so-called “gain spiral.” Gain spirals have been originally described as amplifying loops where cyclic relationships among constructs build on each other positively over time (Lindsley et al., 1995). More recently, this notion has been embedded in the motivational process of the Job Demands-Resources Model (JD-R; Bakker and Demerouti, 2007) in order to explain the reciprocal relationship between resources, work engagement and positive job outcomes, e.g., job satisfaction. In this regard, the interplay between these components is defined as highly dynamic: the motivational process unfolds across time, thus suggesting the occurrence of feedback and feed-forward loops (Salanova et al., 2010). Accordingly, such dynamic interplay may explain the reciprocal and self-perpetuating influence between job satisfaction and work engagement across time. In addition, the positive cycle between work engagement and job satisfaction could contribute to explaining the relative stability over time recorded by job satisfaction in previous research (e.g., Pulakos and Schmitt, 1983; Newton and Keenan, 1991; Schaubroeck et al., 1996; Dormann and Zapf, 2001; Kinicki et al., 2002). Moreover, it is important to highlight that past research has shown that the observed stability of job satisfaction decreases with length of time and as individuals change occupations or employers, suggesting that job satisfaction is influenced by situational factors (Dormann and Zapf, 2001). In line with these assumptions, Giorgi et al. (2015) found that job satisfaction is influenced by dimensions of work-related stress such as job demands and lack of job control, bullying and economic stressors, such as non-employability and fear of the crisis. For this reason, we hypothesized that job demands and age have a moderator effect on the relationship between job satisfaction and work engagement.

### Job Satisfaction and Work Engagement

Job satisfaction is defined as “the extent to which people like (satisfaction) or dislike (dissatisfaction) their jobs” (Spector, 1997, p. 2). It is a result of individuals’ perception and evaluation of their job, influenced by their own unique needs, values and expectations, which they regard as being important to them. The consequences of job satisfaction include better performance and a reduction in withdrawal and counter-productive behavior (Morrison, 2008). According to the Job Demands-Resources Model (JD-R), job satisfaction is a job outcome of the *motivational process* that refers to the effect job resources have on positive outcomes such as job performance, employees’ health, and job satisfaction, through the meditational role of work engagement (Schaufeli and Taris, 2013). Work engagement is considered “as a positive, fulfilling, work-related state of mind that is characterized by vigor, dedication, and absorption” (Schaufeli et al., 2002, p. 74). Engaged employees tend to be more resilient and able to cope effectively with problems, and they are characterized by high levels of energy and by the strong capability to invest effort in order to fulfill their jobs. They also show enthusiasm and pride in their work, and tend to be fully absorbed in and deeply concentrated on their tasks and duties (Schaufeli et al., 2002). Following Schaufeli and Bakker (2004), to achieve new resources people have to invest existing ones, and in this sense, engaged workers are in an ideal situation to amplify their resources and consequently produce positive outcomes (Simbula and Guglielmi, 2013). Although, there is strong empirical evidence of the mediation role played by engagement (Schaufeli and Taris, 2013), most research is cross-sectional in nature, and furthermore, the consequences of engagement have been less empirically studied (Halbesleben, 2010). Concerning the relationship between work engagement and job satisfaction, empirical research has found a moderate correlation among constructs, in both cross-sectional (Schaufeli et al., 2008b), and more recently, longitudinal studies (Simbula and Guglielmi, 2013). One possible explanation can be found in the nature of these two affective states. Job satisfaction represents “the positive emotional reactions and attitudes an individual has toward their job” (Faragher et al., 2005, p. 106). Although some authors tend to conceptualize job satisfaction only in terms of the cognitive component, such as the person’s assessment of the job (i.e., Salanova and Schaufeli, 2009), it is actually more likely that some emotional components are also involved in its definition. Following the circumplex model of affect proposed by Russell and Carroll (1999), we can distinguish the affective states as a linear combination of two dimensions: activation/arousal and pleasure. In particular, job satisfaction represents an affective state reflecting a high level of pleasure and low level of arousal. On the contrary, job engagement is characterized by high levels of both pleasure and activation. Given these circumstances, it is not surprising that they are not highly related. Nevertheless, we expect that job satisfaction and work engagement reinforce each other. On one hand, in line with COR theory assumptions, given that job satisfaction is a function not only of the objective properties of a job but also of “the pleasurable emotional state resulting from the appraisal of one’s job as achieving or facilitating one’s job values” (Locke, 1969), we consider it a state which can boost work engagement. On the other hand, work engagement has been demonstrated to predict a variety of positive outcomes in all life domains. For instance, engaged employees show greater organizational commitment and enhanced job performance (Hakanen et al., 2008), they exhibit higher levels of proactivity (Salanova and Schaufeli, 2008) and are reported to demonstrate better in-role and extra-role behaviors (Bakker and Bal, 2010). Moreover, work engagement is related to higher life satisfaction and better mental and physical health (Shimazu et al., 2012). Surprisingly, the relationship between work engagement and job satisfaction is under-researched (Schaufeli et al., 2008a; Alarcon and Lyons, 2011), although some studies do confirm this relationship.

In other words, we expect that employees who are generally highly dedicated to their work most probably experience higher levels of job satisfaction. Equally, it is also possible that satisfied employees could be more prone to identifying with their job and be strongly committed to their tasks. Following the COR theory, therefore, we can postulate the existence of a gain cycle, in which work engagement and job satisfaction reinforce each other. In this regard, there is initial evidence about the reciprocity of the relationship between these two constructs (Simbula and Guglielmi, 2013).

H1: Work engagement will mediate the relation between job satisfaction over time.

As outlined in the introduction, stress is greatly increasing among employees. Workers have to cope with tight deadlines, processing and memorizing simultaneously different types of information, and many other cognitively exhausting tasks. However, job demand is not only a potentially draining stressor but also a challenging one. According to the differentiation between “challenge” and “hindrance” stressors (LePine et al., 2005), work characteristics can give rise to different reactions in employees. The authors maintain that some stressors, such as role conflict and ambiguity, called hindrance stressors, are related to fatigue and emotional exhaustion because they represent a threat for employees. Workers who face these types of stressors react in terms of greater anxiety and nervousness, and indeed they are related to job dissatisfaction and turnover (LePine et al., 2005; Podsakoff et al., 2007). On the contrary, challenge stressors (i.e., responsibility) are potentially able to promote personal achievement and future gain because employees see them as a “challenge” rather than a threat (Schaufeli and Taris, 2013). Furthermore, on one hand, following the COR theory, the gain cycle becomes more salient in a context of resource loss (i.e., when job demands are high; Hobfoll, 2002) and, on the other hand, following the JD-R model, “job resources gain their motivational potential particularly when employees are confronted with high job demands” (Bakker and Demerouti, 2007, p. 315).

In particular, workload and time pressure are job demands that represent an “overload of demands at work” (Alarcon, 2011, p. 550). Overload corresponds to a challenge stressor, that is potentially able “to promote personal gain or growth, trigger positive emotions and an active or problem-solving style of coping” (LePine et al., 2005, p. 765). Although, prolonged exposure to overload is associated with emotional exhaustion (Alarcon, 2011), this challenge stressor could be also associated with achievement, learning and work attainment. For these reasons, we expect that in workers exposed to a high level of job demands, the relationship between job satisfaction and work engagement will be stronger. Indeed, when employees are satisfied they are more motivated and committed to their jobs, and this increases the resources available for them; this in turn should increase their engagement and identification. This relationship should be particularly strong when workers have to cope with a high job demand, because they will perceive that they have the resources to face the task. Therefore, employees should show greater engagement and involvement when they are committed to mentally demanding jobs.

H2: Job satisfaction T1 will be positively related to work engagement, above all when job demands are high.

We further hypothesized an additional condition under which our mediation model should be established. We tested employees’ age as a moderator of the relationship between work engagement and job satisfaction over time. As stated by McCarthy et al. (2014), there is no full agreement on how old an ‘older’ worker’ is, but the most common choice is to use 50 years as a cut-off, and the main statistics on this topic use 50–55 to conceptualize older workers. Aging is a complex transition in which physical and psychosocial aspects interact and, in this sense, age is an umbrella-variable which captures various changes that take place when people become older (Bal, 2015). Although, a significant number of studies focus on the differences between “younger” and “older” workers (e.g., Bal and Kooij, 2010; James et al., 2011; Kooij et al., 2013), giving the idea that mature workers represent a homogenous group, our intent is to acknowledge that there may be no such a thing as a “typical older worker.” As individuals age, their experiences and attitudes toward work tend to diverge rather than converge (Flynn, 2010; Nakai et al., 2011). Research has shown that some changes in specific personality traits, such as openness to experience or social vitality, could also be present in later years (Roberts et al., 2006). Furthermore, a recent study (von Bonsdorff et al., 2011) showed that mental and physical work strain differed according to the work ability trajectories identified. These results support the idea that older workers experience different conditions also in terms of health.

Moreover, older workers tend to identify more with and be more committed to their own organization, and tend to suppress negative information and events and experience more positive emotions at work (Ng and Feldman, 2007). A strong sense of identification with a group satisfies very important human needs such as belonging and connectedness, which in turn are related to increased levels of job satisfaction and well-being (Ashforth et al., 2008; van Dick and Haslam, 2012). Consistently, there is meta-analytic evidence showing a positive relationship between age and overall job satisfaction (Ng and Feldman, 2007). However, little is known about the antecedents of older workers’ job satisfaction. Recently, Drabe et al. (2015) analyzed the moderating role of age in the relationship between situational job characteristics and job satisfaction, focusing on income, advancement opportunities and security as extrinsic job characteristics, interesting job and independent work as intrinsic job characteristics and good relationships with management and colleagues as social job characteristics. They found that extrinsic and intrinsic job characteristics tend to be more predictive of younger workers’ job satisfaction, while the good relationships with colleagues are more predictive of older workers’ job satisfaction. Specifically, an interesting job, such as new challenges and new or further roles connected to the opportunity to qualify for new jobs or roles, was shown to be less important to older employees. We concentrated on workers aged 50 and older, and expect engagement to increase job satisfaction in the long run, particularly for relatively younger workers. In line with Expectancy Value theory, Kanfer and Ackerman (2004) assume that individuals allocate their resources based on three primary relationships: effort–performance, performance–utility, and effort–utility. First, with increasing age, more effort is needed to achieve high work performances in tasks based on fluid intelligence (i.e., abstract reasoning, attention). Moreover, high performance levels are associated with comparably lower utility levels, because older employees benefit less from an increase in work performance. Finally, higher effort levels are assumed to have lower utility for older employees, who tend to reallocate personal resources from growth goals (e.g., to learn new things) to maintenance and the regulation of loss (e.g., decline in health). In other words, the effect of work engagement on job satisfaction is expected to decline with age. Older workers will tend to be more engaged and more satisfied over time partially due to the intrinsic maturing of the personality during aging (Kanfer and Ackerman, 2004). Indeed, aging is associated with improvement in emotion regulation, particularly in terms of the use of cognitive emotion regulation strategies. Therefore, the effect of work engagement on job satisfaction over time will be greater for relatively younger employees, because older workers will be generally more satisfied also for reasons related to aging itself.

H3: Work engagement will be positively related to job satisfaction T2, above all for relatively younger workers.

**FIGURE 1:**
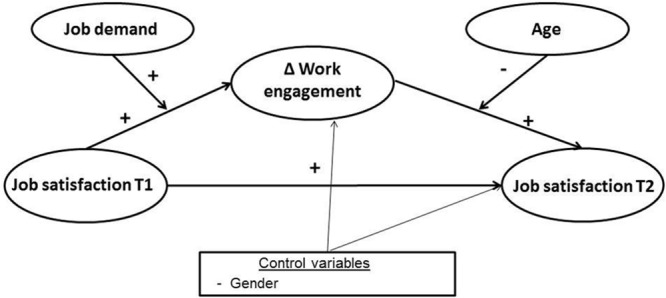
**Full Hypothesized model**.

To summarize, we tested a moderated mediation model (see **Figure 1**), in which job satisfaction will be related to work engagement, which in turn will be related to job satisfaction over time. We also hypothesize 2 moderations by job demands and age. In particular, we expect that this mediational model will be greater for high levels of job demands and for relatively younger employees.

H4: Job satisfaction T1 will be indirectly related to job satisfaction T2 through work engagement, and conditionally to the levels of job demands and age.

## Materials and Methods

### Procedure

Prior to the distribution of the questionnaire, the management of a large local administration body posted a message on the news board of their website informing employees about the aims of the project. The research team then contacted all individuals aged 50 or more via e-mail. The e-mail contained information on the project and the link to an online questionnaire. The survey was designed in accordance with privacy and anonymity regulations (as required by Italian law). The researchers assured the confidentiality of the employees’ responses. 8 months later the research team contacted the participants again, asking them to fill in the questionnaire once more. Times 1 and 2 participants were paired, identifying them by the mean of anonymous codes.

With regard to ethical standards for research, the study adhered to the latest version of the Declaration of Helsinki revised in Fortaleza (World Medical Association [WMA], 2013).

### Participants

The first questionnaire was completed by 1016 workers (64% response rate), the second by 949 employees (60% response rate). All participants were “white collar workers” with permanent and full time contracts. A total of 556 workers (63.5% female) participated in both surveys. The participants’ average age was 55.31 years (*SD* = 3.41), ranging from 50 to 64 years, and with an average organizational tenure of 22.38 years (*SD* = 9.44). After removing missing values at both times, the total sample consisted of 519 workers.

### Measures

Work engagement was assessed through the Italian version of the nine-item Utrecht Work Engagement Scale (Italian validation: Schaufeli et al., 2006; Balducci et al., 2010). The items are grouped into three subscales (three for each dimension): vigor (e.g., “During my work, I feel bursting with energy”), dedication (e.g., “My job inspires me”) and absorption (e.g., “I am immersed in my work”). We followed the recommendation of Schaufeli et al. (2006) and calculated an overall engagement score of the UWES, which we used in the analyses. All items related to dimensions of engagement were scored on a seven-point scale ranging from “0” (never) to “6” (always). The internal consistency reliability (Cronbach’s α) of this scale was 0.90.

To measure Job demands we used eight psychological demands items taken from the Job Content Questionnaire (Karasek, 1985). An example of an item is “I have enough time to get the job done.” The items were scored on a four-point scale ranging from “1” (strongly disagree) to “4” (strongly agree). Cronbach’s alpha was 0.81.

Job satisfaction was assessed with a single item (Wanous et al., 1997). The item was “Overall, how satisfied are you with your job?” and was scored on a five-point scale ranging from “1” (not satisfied at all) to “5” (completely satisfied).

### Data Analyses

To test our hypotheses, we conducted hierarchical multiple regression analyses using the Process macro by Hayes (2013). In particular, we performed the model 21, which makes it possible to test a moderated mediational model in which both the path from the independent variable to mediator and the path from the mediator to dependent variable are moderated by two different variables. To analyze conditional indirect effects, we calculated 95% confidence intervals (CIs) based on bias-corrected bootstrap analyses with 10,000 repetitions.

## Results

Correlations, descriptive statistics, and scale reliabilities are given in **Table 1**.

**Table 1:** Mean, standard deviation, reliabilities (in brackets), and correlations between variables.

	*M*	*SD*	1	2	3	4	5
(1) Age	55.28	3.42	–				
(2) Job demands T1	2.86	0.62	-0.11*	(0.81)			
(3) Work engagement T1	4.53	1.25	-0.01	0.18***	(0.90)		
(4) Work engagement T2	4.42	1.30	-0.02	0.11*	0.70***	(0.90)	
(5) Job satisfaction T1	3.23	0.99	0.03	0.09*	0.55***	0.55***	–
(6) Job satisfaction T2	3.34	1.16	-0.09*	0.02	0.43***	0.58***	0.65***

N = 519. *p < 0.05; ***p < 0.001 (two-tailed).

We checked for non-random distribution of our sample following the analytic strategy suggested by Goodman and Blum (1996). In particular, we calculated a multiple logistic regression in order to ascertain the presence of non-random sampling in our longitudinal data. Since we found that some variables we couldn’t exclude were not non-randomly distributed, we also checked for any differences among variables between participants who filled in both questionnaires (stayers) and those who filled in only the first questionnaire (leavers). We found only two statistical differences on the means and variances of the variables: (i) in particular, those who responded to both surveys (stayers) were relatively younger (*M* = 55.35) than leavers (*M* = 56.06); and (ii) the work engagement variance slightly decreased in stayers compared to whole sample. No differences were found concerning the relation among variables.

**Table 2** presents the detailed results of our hypotheses tests. Variables were centered when necessary to calculate interaction terms. We followed the procedure suggested by Smith and Beaton (2008) ENREF_30, in which changes in the standardized residual scores were used to measure work engagement longitudinally. In particular, by regressing T2 scores of work engagement on the equivalent T1 scores, we obtained the T1–T2 changes in work engagement measured as the standardized residual scores. Positive residual scores indicated an increase in work engagement, while negative scores revealed a decrease. These scores were entered in the model as mediator variable of the relationship between job satisfaction T1 and job satisfaction T2.

**Table 2:** Direct and indirect effects of hypothesized model.

	Δ Engagement (M) *R*^2^ = 0.07***	Job satisfaction T2 (Y) *R*^2^ = 0.49***
	*b (SE)*	*b (SE)*
Gender^a^	-0.03 (0.09)	-0.04 (0.08)
Age	–	0.02* (0.01)
Job satisfaction T1 (JS)	0.24*** (0.04)	0.68*** (0.04)
Job demand T1 (JD)	-0.03 (0.07)	–
Interaction 1 (JS X JD)	0.13* (0.06)	–
Δ Engagement (M)	–	0.30*** (0.04)
Interaction 2 (Age X M)		-0.03* (0.01)

Conditional indirect effects of job satisfaction T1 through Δ Engagement		Job satisfaction T2

		**Point estimate (95% CI)**
Low job demands and younger workers		0.06 (0.01, 0.12)
Low job demands and older workers		0.04 (0.01, 0.09)
High job demands and younger workers		0.12 (0.08, 0.19)
High job demands and older workers		0.07 (0.03, 0.12)

N = 519 (listwise). ^a^0 = male, 1 = female; *p < 0.05; ***p < 0.001. M = mediator variable, Y = dependent variable. Confidence intervals (CIs) of indirect effects based on 10,000 bias corrected bootstrap samples. Unstandardized coefficients are reported.

As shown in the upper part of the table, first of all we computed the model for the mediator (M) work engagement (Δ = standardized residual scores). After checking for gender and in support of hypothesis 1, job satisfaction T1 was significantly related to work engagement (*b* = 0.24, *p* < 0.001). More importantly, and in line with hypothesis 2, the interaction term (computed as the product of job demands T1 and job satisfaction T1 variables) also had a positive and significant relationship to work engagement (*b* = 0.13, *p* < 0.01), over and above the direct effect of job demands itself. This means that job satisfaction T1 increases work engagement particularly for employees who perceived relatively high job demands.

We then calculated the model for the dependent variable (Y) job satisfaction T2. Again, after checking for gender and consistent with hypothesis 3, job satisfaction T1 was a strong predictor of the satisfaction over time both directly (*b* = 0.68, *p* < 0.001) and indirectly through the mediation of work engagement (*b* = 0.30, *p* < 0.001). Finally, and in line with hypothesis 4, after checking for a direct effect of age, also the interaction term, computed as the product of work engagement and age, was negatively and significantly related to job satisfaction T2 (*b* = -0.03, *p* < 0.05), meaning that work engagement increases job satisfaction T2 particularly for younger workers.

**FIGURE 2:**
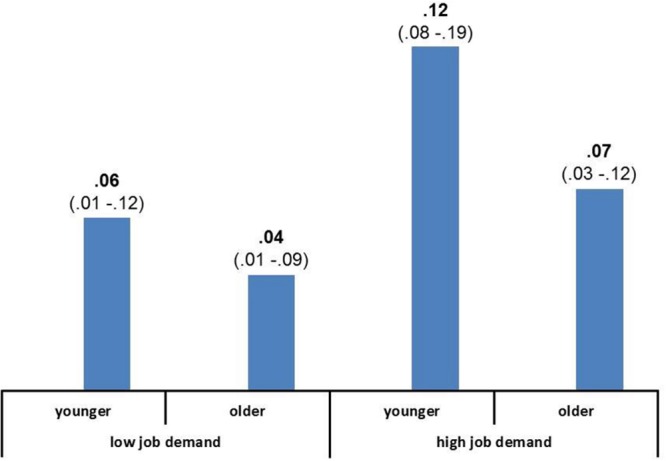
**Indirect effects of the full mediational moderated model.** Confidence intervals of indirect effects based on 10,000 bias corrected bootstrap samples are given in brackets.

We then computed the conditional indirect effects and related bootstrap analyses (see lower part of **Table 2**). In particular, we calculated the indirect effect of job satisfaction T1 on job satisfaction T2 through work engagement, conditionally to the levels of both moderators: job demands and age. As can be seen, all the conditional indirect effects were significant, ranging from 0.04 to 0.12. The sizes of the specific indirect effects at the specific levels of the two moderators and with respective bootstrap CIs are also shown in **Figure 2**. The largest indirect effect size was found for younger employees with relatively high levels of job demands (*b* = 0.12, 95% CI [0.08, 0.19]), while the lowest effect was found for older workers with low levels of job demands (*b* = 0.04, 95% CI [0.01, 0.09]).

## Discussion

A great challenge for the future, as well as for current management, is how to obtain and keep employees motivated and well-performing without jeopardizing their health. This challenge is increased by the great changes that have occurred in the last decades, in particular the constant and predictable aging of the workforce and the increase in job workload (Kompier, 2006; Toossi, 2012). In our study we tested a longitudinal moderated mediation model in which job satisfaction increases over time through an increment in work engagement. Furthermore, we hypothesized that this process was moderated by job demand, and hypothesis 1 was built on the mediating role of engagement in a gain circle of job satisfaction, in this sense examining the mechanism (“how”) on the basis of satisfied employees. However, at the same time it is also important to address the conditions (“when”) under which this mechanism works (hypotheses 2–3). Thus, in this study we tried to answer both how and when questions (Hayes and Preacher, 2013).

The findings confirm our hypotheses. In particular, our results showed that the higher their satisfaction, the more engaged and committed to their job the workers were, which in turn should increase job satisfaction over time, as stated in hypothesis 1. This result, as over mentioned, is in line with the main idea of the COR theory (Hobfoll, 2002) and give reason of a self-perpetuating influence between job satisfaction and work engagement across time. Furthermore, hypothesis 2 stated that such a longitudinal mediation model is moderated by job demands, as confirmed by data. Based on the differentiation between hindrance and challenge stressors proposed by LePine et al. (2005), job demands were considered a challenge stressor, able to increase the relationship between job satisfaction and engagement. Our results showed that satisfied employees are more engaged in their jobs, particularly under the condition of high job demand. In line with the challenge stress approach, they feel able to cope with their demanding job, using such a challenge as an opportunity for growth. Similarly, to previous research that showed how positive affective states, in particular optimism, may foster the capacity to cope with threatening situations (Luthans and Youssef, 2007) and increase work engagement (Xanthopoulou et al., 2009), our findings confirmed that job satisfaction may foster the gain cycle in a challenging workplace. As hypothesized in H3, we also found that age moderates the relationship between work engagement and job satisfaction. As age increases, the effect of work engagement on job satisfaction weakens. Relatively older workers are more satisfied in general, for reasons related to the aging process itself (i.e., maturation, change in personality, etc.), and less attracted by a high level of effort (Kanfer and Ackerman, 2004). In this sense the motivational pattern (engagement – job satisfaction) plays a prominent role especially for relatively younger people. This is important as it suggests that both younger and older workers should be offered more challenge stressors (such as job demands), which could stimulate them, but that the expected effect could be more prominent for relatively younger workers. Our results confirmed that a gain cycle may be found in older workers, although with different trends according to age and job demands. The moderating role of age, which has been confirmed by our research, appears an added value, since understanding the relationship between age, motivation, and engagement is crucial when and where the workforce is growing older (cf. Pitt-Catsouphes and Matz-Costa, 2008). Although this aspect has not been widely investigated, some studies (e.g., James et al., 2011; Guglielmi et al., 2016) underlined an impact of age on employment engagement, though comparing younger vs. older workers. Instead, our study points out that age makes the difference and influences motivation, also within the same group of older workers.

Currently most research on the aging workforce focuses on the difference between older and younger workers, assuming demographic thresholds that usually use cut-off ages between 40 and 50 years. The improvement in health conditions of the population, a shift toward mental rather than physical job demands, and the general increase in the retirement age will probably make future 50-years-old similar to today’s 40-years-old. In general, research showed that important changes occur between age cohorts, even among so-called ‘older’ workers (cf. James et al., 2011; von Bonsdorff et al., 2011), therefore it is important to understand the differences in motivational patterns occurring after the age of 50, as older workers may not be such a homogeneous group. In our sample we decided to use employees aged 50 and over. As seen in the previous references, in developed countries currently the majority of statistics on aging produced by international institutions define ‘older workers’ as those aged from 50 to 55 years and over.

Our results confirm the importance, on one hand, of not considering older workers as a uniform group and, on the other, of taking into account the role played by the gain cycle of resources. As reported by Gorgievski and Hobfoll (2008, p. 5), people “with greater resources are less vulnerable to resource loss and more capable of orchestrating resource gain.” This means that workers with high engagement will not only be more satisfied but will also tend to increase their resources over time, becoming more resilient against stress and adverse job conditions. In our study we observed that this cycle also works in employees over the age of 50.

### Practical Implications

Our findings show that age matters, and that greater consideration should be devoted to age differences in order to design appropriate human resource practices that foster work engagement and satisfaction.

Gain cycle was shown to be moderated by the combined influence of job demand intensity and age: higher job demands and younger age are related to the maximum level of gain cycle, while the same high level of job demands, when associated with older age, appears not to be able to stimulate a similar effect. In terms of managerial implications, job demands represents a potential leverage to increase work engagement, though human resource practices aiming to use this feature should be designed bearing in mind that such a positive and stimulating effect does not impact “younger” and “older” old workers in the same way. Should employers be interested in modifying job design, they could consider increasing job demands (within functional limits) mainly for younger workers: indeed, in principle every worker may be stimulated by a challenging job, although our findings showed that the gain cycle is more likely to be maximized among younger workers. On the other hand, given the role of engagement shown in this study, if employers are unable to make changes in job design, they should bear in mind that other types of interventions are available to improve engagement, such as opportunities for learning and personal development. In fact, training and development activities aimed at developing new skills are important to prevent obsolescence and improve engagement. This is also confirmed in our results by the role of high job demands in work engagement. Stereotypes against older workers should be definitively challenged, and older workers should be offered opportunities to expand their knowledge, as studies show that opportunity for development is related to job satisfaction (Kooij et al., 2010) and the stereotypes held by supervisors on older workers’ avoidance of learning lessen their willingness to train and develop (Van Vianen et al., 2011). Furthermore, it should also be taken into consideration that aging employees are increasingly motivated by intrinsic reasons, so helping others, and the chance to act as mentors could reciprocate in terms of higher engagement.

The current state of studies offers limited evidence on the specific and differential effects of different HRM practices, following different age cohorts within the older workers population (Kooij et al., 2010, 2013). However, our study suggests that age matters as far as the gain cycle of work engagement and positive work outcomes are concerned, therefore it is possible to hypothesize that different practices can be used to motivate middle-aged and older workers. This field of research appears promising and deserves further investigation.

### Limitations

While conducting a longitudinal study increases the power of our inferences, some problems may relate to the sample mortality. This is important because, as outlined by Goodman and Blum (1996, p. 628), “Particular groups of people may be lost in subsequent data collection, resulting in a biased sample or lack of generalizability.” In order to establish the presence or otherwise of non-random sampling, we conducted an attrition analysis. This revealed only two differences between those who participated in both surveys and those who dropped out of the research. Since no differences were found concerning the strength of the relationships among variables, we are confident that subject attrition did not seriously affect our results. In addition to this, our study has several limitations. Firstly, our sample was certainly not representative, and for this reason the generalization of our findings is limited. Future research could replicate our results in different organizations (i.e., private organizations) and in different countries. Another limitation of our study is that only self-reported data were used; future studies could use objective measures to evaluate, for example, job demands (i.e., supervisors’ evaluations). A further limitation concerns the time lag used in this study (8 months). This time lag was chosen for practical convenience. Although, other studies using the same lag time in similar research (see for e.g., Salanova et al., 2011) future studies could select more theoretically grounded intervals, and above all use more measurement points in order to better measure the hypothesized gain cycle.

## Author Contributions

DG, IB, and RC conceptualized the study and chose the theoretical framework. The first version of the introduction was written by LA and RC. LA and MM analyzed the data and wrote the methods and results. DG and MD wrote the discussion and practical implications. All the authors then revised and improved the manuscript several times.

## Conflict of Interest Statement

The authors declare that the research was conducted in the absence of any commercial or financial relationships that could be construed as a potential conflict of interest.

## References

[B1] Alarcon G. M. (2011). A meta-analysis of burnout with job demands, resources, and attitudes. *J. Vocat. Behav.* 79 549–562. 10.1016/j.jvb.2011.03.007

[B2] Alarcon G. M. Lyons J. B. (2011). The relationship of engagement and job satisfaction in working samples. *J. Psychol.* 145 463–480. 10.1080/00223980.2011.584083 21902012

[B3] Arcangeli G. Mucci N. (2009). Health problems in the working occupation of young people in handicraft factories. *G. Ital. Med. Lav. Ergon.* 31 303–306. 19943447

[B4] Ashforth B. E. Harrison S. H. Corley K. G. (2008). Identification in organizations: an examination of four fundamental questions. *J. Manage.* 34 325–374. 10.1177/0149206308316059

[B5] Bakker A. B. Bal P. M. (2010). Weekly work engagement and performance: a study among starting teachers. *J. Occup. Organ. Psych.* 83 189–206. 10.1348/096317909X402596

[B6] Bakker A. B. Demerouti E. (2007). The job demands-resources model: state of the art. *J. Manage. Psychol.* 22 309–328. 10.1108/02683940710733115

[B7] Bal M. P. (2015). “Sustainable careers: enabling older workers to continue working through individualized work arrangements,” in *Handbook of Research on Sustainable Careers* eds De Vos A. Van der Heijden B. (Cheltenham: Edward Elgar) 304–318.

[B8] Bal P. M. Kooij D. (2010). The relations between work centrality, psychological contracts, and job attitudes: the influence of age. *Eur. J. Work Organ. Psychol.* 20 497–523. 10.1080/13594321003669079

[B9] Balducci C. Fraccaroli F. Schaufeli W. B. (2010). Psychometric properties of the Italian version of the Utrecht Work Engagement Scale (UWES-9): a cross-cultural analysis. *Eur. J. Psychol. Assess.* 26 143–149. 10.1027/1015-5759/a000020

[B10] Bedeian A. G. Ferris G. R. Kacmar K. M. (1992). Age, tenure, and job satisfaction: a tale of two perspectives. *J. Vocat. Behav.* 40 33–48. 10.1016/0001-8791(92)90045-2

[B11] Dormann C. Zapf D. (2001). Job satisfaction: a meta-analysis of stabilities. *J. Organ. Behav.* 22 483–504. 10.1002/job.98

[B12] Drabe D. Hauff S. Richter N. F. (2015). Job satisfaction in agin workforces: an analysis of the USA, Japan and Germany. *Int. J. Hum. Resour. Man.* 26 783–805. 10.1080/09585192.2014.939101

[B13] European Commission (2015). *The 2015 Ageing Report Underlying Assumptions and Projection Methodologies.* Brussels: European Commission.

[B14] European Foundation for the Improvement of Living and Working Conditions (2007). *Fourth European Working Conditions Survey.* Dublin: European Foundation for the Improvement of Living, and Working Conditions.

[B15] Faragher E. B. Cass M. Cooper C. L. (2005). The relationship between job satisfaction and health: a meta-analysis. *Occup. Eviron. Med.* 62 105–112. 10.1136/oem.2002.006734 PMC1740950 15657192

[B16] Flynn M. (2010). The United Kingdom government’s “business case” approach to the regulation of retirement. *Ageing Soc.* 30 421–443. 10.1017/S0144686X09990705

[B17] Giorgi G. Shoss M. K. Leon-Perez J. M. (2015). Going beyond workplace stressors: economic crisis and perceived employability in relation to psychological distress and job dissatisfaction. *Int. J. Stress Manag.* 22 137–158. 10.1037/a0038900

[B18] Goodman J. S. Blum T. C. (1996). Assessing the non-random sampling effects of subject attrition in longitudinal research. *J. Manag.* 22 627–652. 10.1177/014920639602200405

[B19] Gorgievski M. J. Hobfoll S. E. (2008). “Work can burn us out or fire us up: conservation of resources in burnout and engagement,” in *Handbook of Stress and Burnout in Health Care* ed. Halbesleben J. R. B. (Hauppauge, NY: Nova Science Publishers) 1–22.

[B20] Guglielmi D. Bruni I. Simbula S. Fraccaroli F. Depolo M. (2016). What drives teacher engagement: a study of different age cohorts. *Eur. J. Psychol. Educ.* 31 323–340. 10.1007/s10212-015-0263-8

[B21] Hakanen J. J. Schaufeli W. B. Ahola K. (2008). The Job Demands-Resources model: a three-year cross-lagged study of burnout, depression, commitment, and work engagement. *Work Stress* 22 224–241. 10.1080/02678370802379432

[B22] Halbesleben J. R. B. (2010). “A meta-analysis of work engagement: relationships with burnout, demands, resources, and consequences,” in *Work Engagement: A Handbook Of Essential Theory and Research* eds Bakker Arnold B. Leiter Michael P. (New York, NY: Psychology Press).

[B23] Hayes A. F. (2013). *Data from: PROCESS: A Versatile Computational Tool for Observed Variable Mediation, Moderation, and Conditional Process Modeling.* Available at: http://afhayes.com/public/process2012.pdf

[B24] Hayes A. F. Preacher K. J. (2013). “Conditional process modeling: using structural equation modeling to examine contingent causal processes,”in *Structural Equation Modeling: A Second Course* eds Hancock G. R. Mueller R. O. (Charlotte, NC: Information Age Publishing).

[B25] Hobfoll S. E. (1989). Conservation of resources: a new attempt at conceptualizing stress. *Am. Psychol.* 44 513–524. 10.1037/0003-066X.44.3.513 2648906

[B26] Hobfoll S. E. (2002). Social and psychological resources and adaptation. *Rev. Gen. Psychol.* 6 307–324. 10.1037/1089-2680.6.4.307

[B27] Hobfoll S. E. (2011). Conservation of resource caravans and engaged settings. *J. Occup. Organ. Psych.* 84 116–122. 10.1111/j.2044-8325.2010.02016.x

[B28] James J. B. McKechnie S. Swanberg J. (2011). Predicting employee engagement in an age-diverse retail workforce. *J. Organ. Behav.* 32 173–196. 10.1002/job.681

[B29] Kalleberg A. L. Loscocco K. A. (1983). Aging, values, and rewards: explaining age differences in job satisfaction. *Am. Sociol. Rev.* 48 78–90. 10.2307/2095146 6838066

[B30] Kanfer R. Ackerman P. L. (2004). Aging, adult development, and work motivation. *Acad. Manag. Rev.* 29 440–458. 10.5465/AMR.2004.13670969

[B31] Karasek R. A. (1985). *Job Content Questionnaire and User’s Guide.* Lowell, MA: University of Massachusetts.

[B32] Kinicki A. J. McKee-Ryan F. M. Schriesheim C. A. Carson K. P. (2002). Assessing the construct validity of the job descriptive index: a review and meta-analysis. *J. Appl. Psychol.* 87 14–32. 10.1037//0021-9010.87.1.14 11916208

[B33] Kompier M. A. (2006). New systems of work organization and workers’ health. *Scand. J. Work Env. Health* 32 421–430. 10.5271/sjweh.1048 17173199

[B34] Kooij D. T. Guest D. E. Clinton M. Knight T. Jansen P. G. Dikkers J. S. (2013). How the impact of HR practices on employee well-being and performance changes with age. *Hum. Resour. Manag. J.* 23 18–35. 10.1111/1748-8583.12000

[B35] Kooij D. T. Jansen P. G. Dikkers J. S. De Lange A. H. (2010). The influence of age on the associations between HR practices and both affective commitment and job satisfaction: a meta analysis. *J. Organ. Behav.* 31 1111–1136. 10.1002/job.666

[B36] LePine J. A. Podsakoff N. P. LePine M. A. (2005). A meta-analytic test of the challenge stressor hindrance stressor framework: an explanation for inconsistent relationships among stressors and performance. *Acad. Manag. Rev.* 48 764–775. 10.5465/AMJ.2005.18803921

[B37] Lindsley D. H. Brass D. J. Thomas J. B. (1995). Efficacy-performing spirals: a multilevel perspective. *Acad. Manag. Rev.* 20 645–678. 10.2307/258790

[B38] Locke E. A. (1969). What is job satisfaction? *Organ. Behav. Hum. Perf.* 4 309–336. 10.1016/0030-5073(69)90013-0

[B39] Luthans F. Youssef C. M. (2007). Emerging positive organizational behavior. *J. Manag.* 33 321–349. 10.1177/0149206307300814

[B40] McCarthy J. Heraty N. Cross C. Cleveland J. N. (2014). Who is considered an “older worker”? Extending our conceptualization of “older” from an organizational decision maker perspective. *Hum. Resour. Manag. J.* 24 374–393. 10.1111/1748-8583.12041

[B41] Morrison R. (2008). Negative relationships in the workplace: associations with organizational commitment, cohesion, job satisfaction and intention turnover. *J. Manage. Organ.* 14 330–344. 10.1017/S1833367200003126

[B42] Nakai Y. Chang B. Snell A. Fluckinger C. (2011). Profiles of mature job seekers: connecting needs desires to work characteristics. *J. Organ. Behav.* 32 155–172. 10.1002/job.697

[B43] Newton T. Keenan T. (1991). Further analyses of the dispositional argument in organizational behavior. *J. Appl. Psychol.* 76:781 10.1037/a0030939

[B44] Ng T. W. Feldman D. C. (2007). Organizational embeddedness and occupational embeddedness across career stages. *J. Vocat. Behav.* 70 336–351. 10.1016/j.jvb.2006.10.002

[B45] Pitt-Catsouphes M. Matz-Costa C. (2008). *The Multi-Generational Workforce: Findings from the Age & Generations Study.* Chestnut Hill, MA: Sloan Center on Aging & Work at Boston College.

[B46] Podsakoff N. P. LePine J. A. LePine M. A. (2007). Differential challenge stressor-hindrance stressor relationships with job attitudes, turnover intentions, turnover, and withdrawal behavior: a meta-analysis. *J. Appl. Psychol.* 92 438–454. 10.1037/0021-9010.92.2.438 17371090

[B47] Pulakos E. D. Schmitt N. (1983). A longitudinal study of a valence model approach for the prediction of job satisfaction of new employees. *J. Appl. Psychol.* 68 307–312. 10.1037/0021-9010.68.2.307

[B48] Roberts B. W. Walton K. E. Viechtbauer W. (2006). Patterns of mean-level change in personality traits across the life course: a meta_analysis of longitudinal studies. *Psychol. Bull.* 132 3–27. 10.1037/0033-2909.132.1.1 16435954

[B49] Russell J. A. Carroll J. M. (1999). On the bipolarity of positive and negative affect. *Psychol. Bull.* 125 3–30. 10.1037/0033-2909.125.1.3 9990843

[B50] Salanova M. Llorens S. Schaufeli W. B. (2011). Yes, I can, I feel good, and I just do it! On gain cycles and spirals of efficacy beliefs, affect, and engagement. *Appl. Pyschol. Int. Rev.* 60 255–285. 10.1111/j.1464-0597.2010.00435.x

[B51] Salanova M. Schaufeli W. (2009). *El Engagement en el Trabajo: Cuando el Trabajo se Convierte en Pasión.* Madrid: Alianza Editorial.

[B52] Salanova M. Schaufeli W. B. (2008). A cross-national study of work engagement as a mediator between job resources and proactive behaviour. *Int. J. Hum. Resour. Manag.* 19 116–131. 10.1080/09585190701763982

[B53] Salanova M. Schaufeli W. B. Xanthopoulou D. Bakker A. B. (2010). “The gain spiral of resources and work engagement: sustaining a positive worklife,” in *Work Engagement: A Handbook of Essential Theory and Research* eds Bakker A. B. Leiter M. P. (New York, NY: Psychology Press) 118–131.

[B54] Schaubroeck J. Ganster D. C. Kemmerer B. (1996). Research Does trait affect promote job attitude note stability? *J. Organ. Behav.* 17 191–196. 10.1002/(SICI)1099-1379(199603)17:2<191::AID-JOB777>3.3.CO;2-O

[B55] Schaufeli W. B. Bakker A. B. (2004). Job demands, job resources, and their relationship with burnout and engagement: a multi-sample study. *J. Organ. Behav.* 25 293–315. 10.1002/job.248

[B56] Schaufeli W. B. Bakker A. B. Salanova M. (2006). The measurement of work engagement with a short questionnaire a cross-national study. *Educ. Psychol. Meas.* 66 701–716. 10.1177/0013164405282471

[B57] Schaufeli W. B. Salanova M. González-Romá V. Bakker A. B. (2002). The measurement of engagement and burnout: a two sample confirmatory factor analytic approach. *J. Happiness Stud.* 3 71–92. 10.1023/A:1015630930326

[B58] Schaufeli W. B. Taris T. W. (2013). “A critical review of the job demands-resources model: implications for improving work and health,” in *Bridging Occupational, Organizational and Public Health: A Transdisciplinary Approach* eds Bauer G. F. Hämmig O. (Dordrecht: The Netherlands: Springer) 43–68.

[B59] Schaufeli W. B. Taris T. W. Bakker A. B. (2008a). “It takes two to tango: workaholism is working excessively and working compulsively,” in *The long Work Hours Culture. Causes, Consequences and Choices* eds Burke R. J. Cooper C. L. (Bingley: Emerald) 203–226.

[B60] Schaufeli W. B. Taris T. W. van Rhenen W. (2008b). Workaholism, burnout, and work engagement: three of a kind or three different kinds of employee well-being? *Appl. Psychol.* 57 173–203. 10.1111/j.1464-0597.2007.00285.x

[B61] Shimazu A. Schaufeli W. B. Kubota K. Kawakami N. (2012). Do workaholism and work engagement predict employee well-being and performance in opposite directions? *Ind. Health* 50 316–321. 10.2486/indhealth.MS1355 22673364

[B62] Simbula S. Guglielmi D. (2013). I am engaged, I feel good, and I go the extra-mile: reciprocal relationships between work engagement and consequences. *Rev. Psicol. Trab. Organ.* 29 117–125. 10.5093/tr2013a17

[B63] Sloan Center on Aging and Work at Boston College (2011). *Data from: Effects of “Old-Developed” versus “Young Developing” Country Type and Age-Related Factors on Work Engagement, Job Satisfaction, & Organizational Commitment.* (2011) Available at http://www.bc.edu/content/dam/files/research_sites/agingandwork/pdf/publications/GOT_CR_Sumary.pdf

[B64] Smith P. Beaton D. (2008). Measuring change in psychosocial working conditions: methodological issues to consider when data are collected at baseline and one follow-up time point. *Occup. Environ. Med.* 65 288–296. 10.1136/oem.2006.032144 18349161

[B65] Spector P. E. (1997). *Job Satisfaction: Application, Assessment, Causes, and Consequences.* Thousand Oaks, CA: Sage.

[B66] Toossi M. (2012). Projections of the labor force to 2050: a visual essay. *Mon. Labor Rev.* 135 3–16.26005226

[B67] United Nations (2007). *World Economic and Social Survey 2007.* New York, NY: Development in an Ageing World, United Nations Publication.

[B68] van Dick R. Haslam S. A. (2012). “Stress and well-being in the workplace: support for key propositions from the social identity approach,” in *The Social cure: Identity, Health, and Well-Being* eds Jetten J. Haslam C. Haslam S. A. (London: Psychology Press) 1–27.

[B69] Van Vianen A. E. Dalhoeven B. A. De Pater I. E. (2011). Aging and training and development willingness: employee and supervisor mindsets. *J. Organ. Behav.* 32 226–247. 10.1002/job.685

[B70] von Bonsdorff M. E. Kokko K. Seitsamo J. von Bonsdorff M. B. Nygård C. H. Ilmarinen J. (2011). Work strain in midlife and 28-year work ability trajectories. *Scand. J. Work. Env. Health* 37 455–463. 10.5271/sjweh.3177 21695372

[B71] Wanous J. P. Reichers A. E. Hudy M. J. (1997). Overall job satisfaction: how good are single item measures? *J. Appl. Psychol.* 82 247–252. 10.1037/0021-9010.82.2.247 9109282

[B72] World Medical Association [WMA] (2013). World Medical Association Declaration of Helsinki ethical principles for medical research involving human subjects. *J. Amr. Med. Assoc.* 310 2191–2194. 10.1001/jama.2013.281053 24141714

[B73] Xanthopoulou D. Bakker A. B. Demerouti E. Schaufeli W. B. (2009). How job and personal resources influence work engagement and financial returns: a diary study in a Greek fast-food company. *J. Occup. Organ. Psych.* 82 183–200. 10.1348/096317908X285633

